# Asymptomatic autoamputation of ovary and fallopian tube in a 2 days newborn: a case report

**DOI:** 10.11604/pamj.2021.39.278.30553

**Published:** 2021-08-27

**Authors:** Dimitrios Godosis, Vassilis Lambropoulos, Vasileios Mouravas, Eleftheria Massa, Kleanthis Anastasiadis, Chrysostomos Kepertis, Ioannis Spyridakis

**Affiliations:** 1 2^nd^ Paediatric Surgery Department, Aristotle University of Thessaloniki, “Papageorgiou” General Hospital, Thessaloniki, Greece,; 2 Department of Pathology, “Papageorgiou” General Hospital, Thessaloniki, Greece

**Keywords:** Floating ovary, torsion, autoamputation, neonate, case report

## Abstract

Automatic amputation of the ovary represents a rather uncommon condition. Especially asymptomatic autoamputation is an even more unusual laparoscopic finding. We hereby present a case of a 2-days´-old infant with a prenatal ultrasound (US) diagnosis of a cystic mass, laparoscopically proved as an amputated right adnexa. The female infant was asymptomatic and had normal laboratory exams, including hormone levels, according to her age. The infant was managed surgically, as the size of the cystic lesion, both prenatally and postnatally was indicative of surgical intervention. Careful monitoring is critical for the management of cystic lesions diagnosed prenatally. Although rare, the suspicion of an auto-amputated ovary has to be risen during diagnostic approach of infants with adnexal cysts, especially when these lesions are supposed to “wander” during imaging examinations, and also taking into account the size of the lesion in order for a final approach and management to be established.

## Introduction

Regarding ovarian amputation, it is indeed a rare complication of a detorsed adnexa or ovarian cyst prenatally or postnatally. Asymptomatic cases are manifested as incidental findings during laparoscopy for other surgical reasons, or even as floating intra-abdominal mass during routine ultrasound (US) prenatal examination.

## Patient and observation

**Figure 1:**
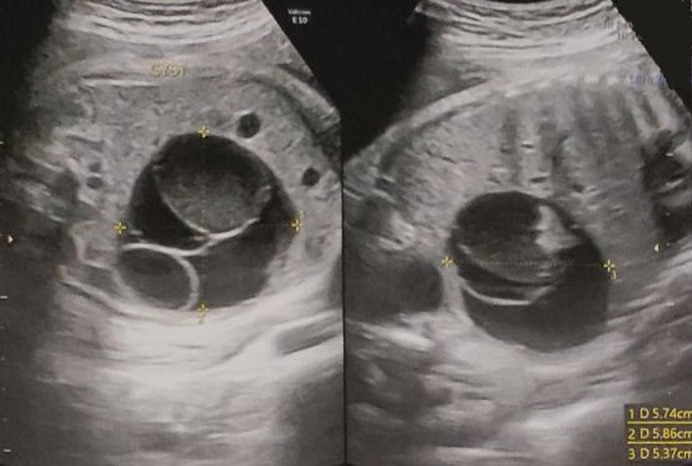
prenatally diagnosed abdominal cystic lesion, measuring 58x57x54mm in US examination

**Patient information:** a 2-days-old female was admitted to our Pediatric Surgery Department with a prenatally diagnosed intra-abdominal mass. Regarding the baby´s history, she had a free paternal and maternal medical history. During the 34^th^ week of gestation she was prenatally diagnosed with an abdominal cystic lesion measuring 58x57x54mm in US examination (Figure 1). It is remarkable that no such lesion was mentioned to previous routine embryonic ultrasound examinations during the prenatal period.

**Clinical findings:** after a full-term gestation, and aging one day, the neonate had an uneventful clinical condition, was afebrile, with normal development, no signs of pain, and with normal bowel sounds and bowel habits according to her age. On palpation, no abdominal mass and no abdominal distension were noted.

**Figure 2:**
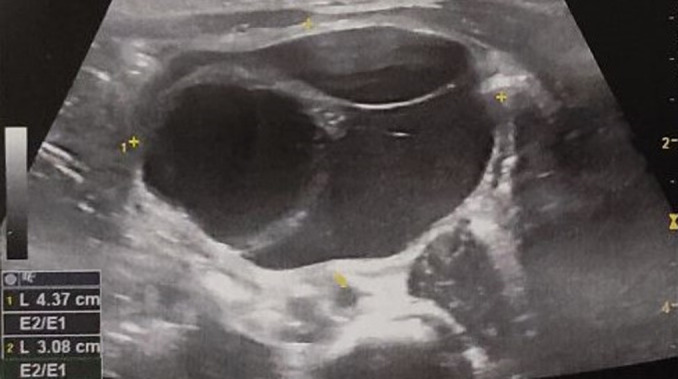
postnatal US examination, during the first day of life, revealing the prenatally diagnosed cystic lesion, measuring 50x30x43mm and based at the left lower abdomen, with no signs of vascularization, characterized by septations with internal echoes and a fluid-debris level

**Diagnostic assessment:** the neonate had a new US exam during her first day of life, which revealed the above mentioned cystic lesion, measuring 50x30x43mm and based at the left lower abdomen, with no signs of vascularization (Figure 2). The cystic lesion was characterized by septations with internal echoes and a fluid-debris level which was moving along with the infant´s position changes. Both prenatal and postnatal imaging findings were indicative of surgical intervention. During her admission in our department, the infant had a preoperative preparation, with routine laboratory exams, including α-fetoprotein (α-FP) and beta human chorionic gonadotropin (b-HCG) measured within normal limits, according to her age.

**Diagnosis:** after the completion of laboratory and imaging examinations, a preoperative diagnosis of an intra-abdominal cystic lesion was established and postoperative histopathology examinations would indicate the final diagnosis and treatment.

**Figure 3:**
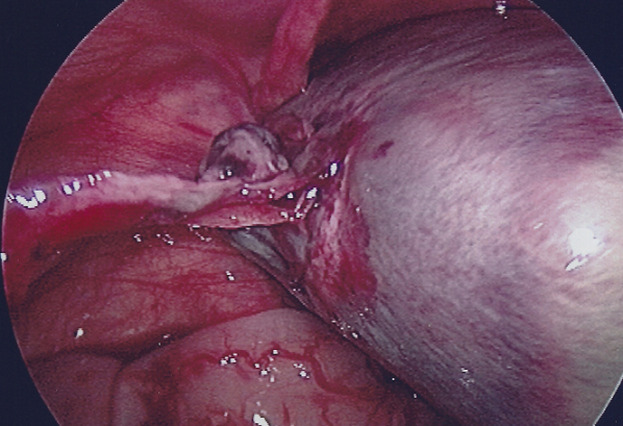
direct visualization via laparoscopic approach revealed a spherical mass, freely floating inside the peritoneal cavity, with an attachment to a small portion of the distal part of the *omentum*

**Figure 4:**
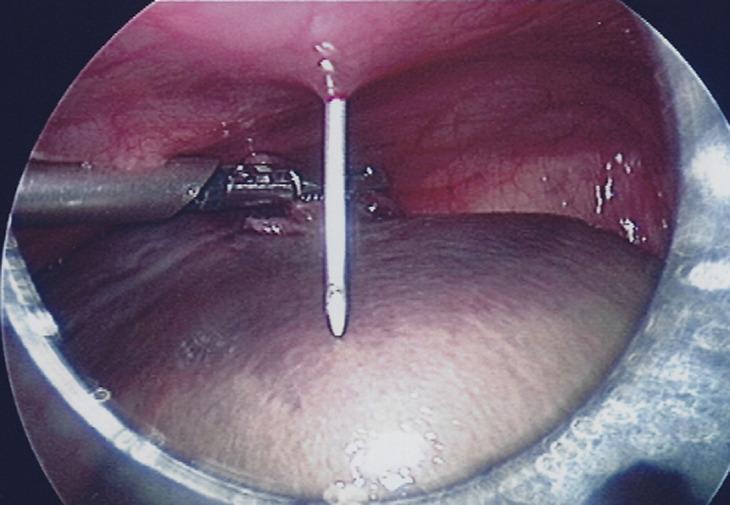
cystic lesion was recognized intra-operatively as the auto amputated right ovary, accompanied with the distal part of fallopian tube; it was punctured with a needle inserted through the abdominal wall, draining a brownish liquid

**Therapeutic intervention:** the infant underwent a laparoscopy exploration. It is noteworthy that after induction to anesthesia, a mass seemed to wander inside the abdomen on palpation. Through an umbilical incision, a 5mm port was inserted and a 30^o^ camera through it. Via a direct laparoscopic vision, two other ports, one in the right lower abdomen and the other in the left lower abdomen were inserted. Laparoscopy revealed a spherical floating mass inside the peritoneal cavity, with an attachment to a small portion of the distal part of the omentum (Figure 3). Careful inspection of the inner genitalia also revealed that this mass originated from the right adnexa, as the cystic lesion was auto-amputated across the half of the length of the fallopian tube. As a result, the free-floating mass included the right ovary and the distal half of the fallopian tube. The cystic lesion was punctured with a needle inserted through the abdominal wall (Figure 4), via direct visualization, draining a brownish liquid. Finally, the mass was exerted through the umbilicus and was sent for histopathology analysis.

**Figure 5:**
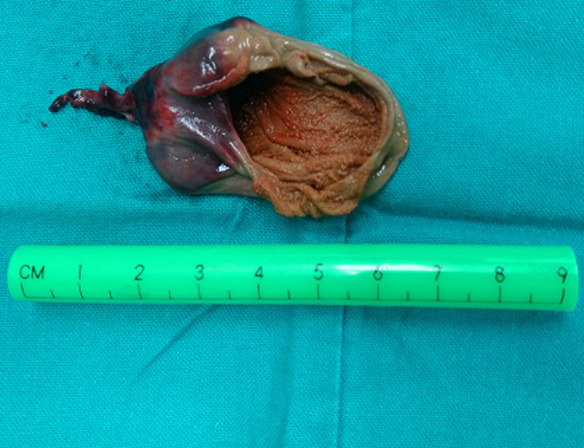
pathology department received a macroscopically cystic specimen measuring 4.8cm in its maximum diameter

**Figure 6:**
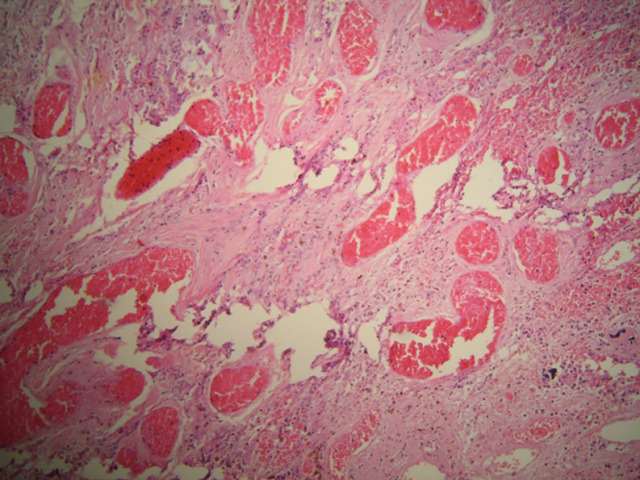
light microscopy of hematoxylin and eosin-stained specimen in a 40x magnification revealed an outer layer of fibrous tissue, lined with flattened cells. Hemorrhagic elements and also stretched and congested vessels were noted in a diffuse pattern

**Follow up and outcomes:** histopathology revealed a macroscopically cystic lesion with a maximum diameter of 4.8cm (Figure 5). Microscopic characteristics included an outer layer of fibrous tissue lined with flattened cells. Hemorrhagic elements and also stretched and congested vessels were noted in a diffuse pattern (Figure 6). No signs of malignancy were observed. The infant had an uneventful postoperative period, according to follow up visits.

## Discussion

Auto-amputation of the ovary is a rather uncommon condition among pediatric population, predominantly emerging from a cystic lesion of the ovary. It is suggested that a certain factor for ovarian cyst development is the augmented stimulation by maternal and placental hormones. Maternal history of diabetes, rhesus immunization, or increased permeability of placenta to b-HCG are also risk factors for such a pathologic entity [1]. According to recent literature, regarding a mini-review of adnexal auto-amputation in a series of totally 94 patients aging 1 day-old to 18 years old, it is reported that 15 of them, aging 1 day to 12 months at the age of treatment, were antenatally diagnosed [2]. Only two of these cases represented free-floating abdominal cyst as a preoperative diagnosis [2, 3]. It is also concluded that common etiology represents a prenatal or postnatal torsion of an ovarian cyst and subsequent devascularization. The mechanical theory is commonly suggested, concluding that chronic torsion and compromise of blood flow lead to auto-amputation. The amputated ovary can sometimes be attached to adjacent structures, such as the abdominal wall, sigmoid colon or small intestine. However, there is also a possibility that a free-floating abdominal mass occurs, a “wandering ovary” with no attachments.

In terms of antenatal and postnatal management, it is undoubted that imaging evaluation with US scan remains a gold standard. Antenatal US examination has proved, due to its widespread use, a prompt and reliable tool for diagnosis and management. The fact that it represents a non-interventional and quite inexpensive method is also in favor for the implementation of US in such small ages of patients. The proposed algorithm for management of these lesions depends predominantly on the lesion´s size. Regarding antenatal investigation, ovarian cystic lesions larger than 2cm in a fetus have to be put in observation, because they are considered abnormal [4, 5]. A cut-off point of 4cm in diameter in postnatal imaging examination is used for decision making. If the lesion is larger than 4 cm in diameter postnatally in repeated imaging examinations, surgical excision is recommended [2].

In our case the cystic lesion was intraoperatively proved as an auto-amputated ovary on the basis of a prenatal torsion. It is also important that a laparoscopic approach was selected, as this allows intraoperative diagnostic capabilities, such as inspection of peritoneal cavity or even biopsy and also offers alternative therapeutic measures, such as aspiration, with preservation of adnexa, if it is proved viable, with the least interventional operative method.

However, even if the diagnosis of free-floating adnexa has not been preoperatively established, surgical management should be considered in all cases of adnexal cystic lesions larger than 4cm in postnatal measurements, as these are prone to torsion and hemorrhage. Furthermore, possible reimplantation of auto-amputated adnexa into *peritoneum*or *omentum*may lead to malignant transformation, as reported [3, 6, 7]. Therefore, a watch-and-wait approach is not considered appropriate under the above circumstances, because only lesions smaller than 4cm may spontaneously resolve in follow-up examinations.

## Conclusion

Management of cystic lesions of inner genitalia in neonates and infants remain a challenging topic for the pediatric surgeon. Although rare, even the suspicion of an auto-amputated ovary has to be risen during diagnostic approach of infants with adnexal cysts, especially when these lesions are supposed to “wander” during imaging examinations, and also taking into account the size of the lesion in order for a final management approach to be established.
